# Serum GDF-15 Levels Accurately Differentiate Patients with Primary Mitochondrial Myopathy, Manifesting with Exercise Intolerance and Fatigue, from Patients with Chronic Fatigue Syndrome

**DOI:** 10.3390/jcm12062435

**Published:** 2023-03-22

**Authors:** Laura Bermejo-Guerrero, Carlos Pablo de Fuenmayor-Fernández de la Hoz, María Paz Guerrero-Molina, Paloma Martín-Jiménez, Alberto Blázquez, Pablo Serrano-Lorenzo, David Lora, Montserrat Morales-Conejo, Irene González-Martínez, Elena Ana López-Jiménez, Miguel A. Martín, Cristina Domínguez-González

**Affiliations:** 1 Neuromuscular Disorders Unit, Department of Neurology, Hospital Universitario 12 de Octubre, 28041 Madrid, Spain; 2 Department of Neurology, Hospital Universitario 12 de Octubre, 28041 Madrid, Spain; 3 Mitochondrial and Neuromuscular Disorders Group, Hospital 12 de Octubre Health Research Institute (imas12), 28041 Madrid, Spain; 4 Biomedical Network Research Centre on Rare Diseases (CIBERER), Instituto de Salud Carlos III, 28029 Madrid, Spain; 5 Clinical Research Unit, Hospital 12 de Octubre Research Institute (imas12), 28041 Madrid, Spain; 6 Department of Statistics and Data Science, Facultad de Estudios Estadísticos, Universidad Complutense de Madrid, 28040 Madrid, Spain; 7 Centro de Investigación Biomédica en Red de Epidemiología y Salud Pública (CIBERESP), 28029 Madrid, Spain; 8 Internal Medicine Department, Hospital Universitario 12 de Octubre, 28041 Madrid, Spain; 9 Congenital Metabolic Defects Group, Hospital Universitario 12 de Octubre, 28041 Madrid, Spain; 10 Clinical Biochemistry Department, Hospital Universitario 12 de Octubre, 28041 Madrid, Spain; 11 Genetics Department, Hospital Universitario 12 de Octubre, 28041 Madrid, Spain

**Keywords:** mitochondrial myopathy, chronic fatigue syndrome, GDF-15, differential diagnosis

## Abstract

Primary mitochondrial myopathies (PMM) are a clinically and genetically highly heterogeneous group that, in some cases, may manifest exclusively as fatigue and exercise intolerance, with minimal or no signs on examination. On these occasions, the symptoms can be confused with the much more common chronic fatigue syndrome (CFS). Nonetheless, other possibilities must be excluded for the final diagnosis of CFS, with PMM being one of the primary differential diagnoses. For this reason, many patients with CFS undergo extensive studies, including extensive genetic testing and muscle biopsies, to rule out this possibility. This study evaluated the diagnostic performance of growth differentiation factor-15 (GDF-15) as a potential biomarker to distinguish which patient with chronic fatigue has a mitochondrial disorder. We studied 34 adult patients with symptoms of fatigue and exercise intolerance with a definitive diagnosis of PMM (7), CFS (22), or other non-mitochondrial disorders (5). The results indicate that GDF-15 can accurately discriminate between patients with PMM and CFS (AUC = 0.95) and between PMM and patients with fatigue due to other non-mitochondrial disorders (AUC = 0.94). Therefore, GDF-15 emerges as a promising biomarker to select which patients with fatigue should undergo further studies to exclude mitochondrial disease.

## 1. Introduction

Mitochondrial diseases can present a wide range of symptoms and affect different organ systems, including the central and peripheral nervous systems. In some cases, the symptoms are limited to skeletal muscle (i.e., primary mitochondrial myopathies, PMM) and can include ptosis, ophthalmoparesis, progressive weakness, or rhabdomyolysis episodes, as previously stated in the international consensus definition [1]. However, some patients may have an almost normal neurological examination but experience symptoms such as exercise intolerance, myalgias, and muscle fatigue, often described as a feeling of “tiredness” or “lack of energy” [2,3]. This presentation can be mistaken for chronic fatigue syndrome (CFS) due to the overlap in symptoms. CFS is a complex and debilitating condition characterized by persistent fatigue that is not improved by rest and is accompanied by a range of other symptoms. The exact cause of CFS is unknown and is thought to be a combination of genetic, environmental, and psychological factors. Symptoms of CFS include severe fatigue, muscle weakness, poor sleep, headaches, joint pain, and difficulty with memory and concentration. There is currently no specific test or biomarker for the diagnosis of CFS [4,5]. It is typically diagnosed based on the presence of symptoms and the exclusion of other potential causes. In this regard, mitochondrial disease is one of the main differential diagnoses. Therefore, many patients undergo extensive diagnostic testing to exclude this possibility. The low sensitivity and specificity of classical laboratory studies, such as creatine kinase (CK) or lactate levels, to detect PMM further complicate the clinical scenario [6]. Furthermore, even genetic testing for mitochondrial myopathies can remain elusive when performed on tissues such as blood and urine, eventually leading to more invasive studies, including muscle biopsy, to obtain morphologic and/or genetic evidence to confirm or exclude this diagnosis [7]. 

The need for sensitive and specific biomarkers to aid in diagnosing underlying mitochondrial disorders in patients experiencing fatigue and exercise intolerance is of paramount importance. The availability of such biomarkers would assist in identifying patients who would benefit from more advanced or invasive studies. Concerning this, fibroblast growth factor 21 (FGF-21) and growth differentiation factor 15 (GDF-15) have been thoroughly studied, and extensive research supports their potential utility as diagnostic biomarkers [6,8]. According to recent reports, GDF-15 outperforms FGF-21 based on diagnostic sensitivity, diagnostic odds ratio (OR), and receiver operating characteristic (ROC) curves, with no increased benefit of combining both for ROC curve analysis [6]. Furthermore, GDF-15 is indicative of mitochondrial disease regardless of the phenotype, whereas FGF-21 appears to better identify patients with muscle manifestations [6].

In this study, we aimed to assess the diagnostic performance of GDF-15 in patients with mitochondrial disease that presents only as fatigue and exercise intolerance and to distinguish them from those with chronic fatigue syndrome.

## 2. Materials and Methods

### 2.1. Patients

We obtained data from 34 adult patients with fatigue and exercise intolerance symptoms treated at the Neuromuscular Disorders Unit of the Hospital Universitario 12 de Octubre in Madrid, a national reference center of rare neuromuscular diseases in Spain. The patients comprised 7 with mitochondrial myopathy, 5 with a non-mitochondrial disorder, and 22 with CFS. The diagnosis of mitochondrial disease and non-mitochondrial disorders was confirmed through genetic testing. Diagnostic studies were conducted as part of routine clinical practice for patients diagnosed with CFS to rule out other conditions, including muscle biopsies for all patients and genetic testing for 18 patients (Table 1).

### 2.2. Muscle Biopsy

Histological, histochemical, and immunohistochemical stains after muscle biopsies were performed according to standard protocols as part of the routine diagnostic workup. Genetic tests performed on mtDNA extracted from muscle included: Southern blot and long-range PCR to exclude single or multiple mtDNA deletions (7 patients), a panel of the 20 most common pathogenic mtDNA variants (Table S1) and the c.1399G>A (p.Ala467Thr) *POLG* mutation (13 patients), or complete mtDNA sequencing (2 patients).

### 2.3. Genetic Analysis

Genetic tests performed in blood included next-generation sequencing (NGS) of a panel of specific genes associated with metabolic myopathies (5 patients) and a panel of genes related to mtDNA maintenance mitochondrial disorders (3 patients) (Table S1).

### 2.4. GDF-15 Analysis

Circulating GDF-15 was assessed in serum by a quantitative electrochemiluminescence immunoassay in all patients (Elecsys GDF-15; Roche Diagnostics, Basel, Switzerland).

Reference values (pg/mL) were provided by the kit’s manufacturer, according to age (median, 95th percentile): 30–<40 (500, 852), 40–<50 (614, 1229), 50–<60 (757, 1466), 60–<70 (866, 1476), >70 (1060, 2199).

### 2.5. Other Complementary Tests

Additional evaluations and complementary studies were conducted according to standard protocols, and their results were reviewed retrospectively using medical records. When performed, we collected information on CK and lactate levels, respiratory chain activity, and electrophysiology.

Informed consent was obtained according to the Hospital 12 de Octubre Ethics Committee requirements.

### 2.6. Statistical Data Analysis

Measurable and categorical variables were described with the median, interquartile range, or frequency distribution. Continuous variables were compared with the Mann–Whitney U test. The area under the ROC curve was estimated to evaluate the discrimination ability of the GDF-15 test, and the ROC plots were generated. All estimations were reported with 95% confidence intervals (CI). A *p*-value < 0.05 was considered statistically significant. All statistical analyses were performed using GraphPad PRISM 6 (GraphPad Software, La Jolla, CA, USA) and Stata 16 software (StataCorp. 2019. Stata Statistical Software: Release 16. College Station, TX: StataCorp LLC., Houston, TX, USA).

## 3. Results

The clinical features identified and results of complementary studies on the 34 patients are summarized in Table 1. The mean age (SD) was similar in the three studied groups of patients: 44.4 (9.9) for the group with PMM, 47.1 (8.8) for patients with CFS, and 44.8 (11.1) for patients with other diagnoses. Females represented 57%, 40%, and 86% of each group, respectively. Their representation was not likely to considerably affect the comparison of GDF-15 mean concentrations between the different study groups, as previously published works have shown that there is no significant sex-related difference in its levels [6,9]. 

All PMM patients had persistently elevated CK values, whereas none of the CFS patients did. Only two patients with PMM had mildly elevated lactate levels (28%), and none in the group of patients with CFS. 

Muscle biopsies were obtained in all patients except one from the PMM group. Histologic findings were consistent with an underlying mitochondrial disorder in all confirmed PMM cases, with ragged-red fibers (RRF) and cytochrome C oxidase (COX)-negative fibers present to a variable extent. Respiratory chain complex activity was abnormal in only two PMM patients (28%). In the CFS group, the muscle biopsies did not show any histologic features suggestive of mitochondrial oxidative phosphorylation (OXPHOS) dysfunction. They were completely normal in all but three patients who presented mild non-diagnostic non-specific changes. Only one patient with CFS showed alteration in the respiratory chain activity (a mild complex I deficiency), with normal results in the rest of her complementary tests, including extensive genetic testing (patient 22). 

Electrophysiological studies were available in three patients with PMM, three with a non-mitochondrial disorder, and seventeen patients with CFS. Abnormal findings were seen in all available studies from PMM patients, with one reported as myopathic and the other two as neurogenic (related to polyneuropathy in patient 3 and chronic lumbar radiculopathy in patient 6). Patients with a non-mitochondrial disorder showed normal results in two cases. One patient with a final diagnosis of a congenital myasthenic syndrome, showed a pathologic decrement in repetitive nerve stimulation (RNS) consistent with a pre-synaptic neuromuscular transmission defect. Regarding CFS patients, 5 out of 17 electrophysiological studies (29.4%) were deemed myopathic, and the rest normal (12).

**Figure 1:**
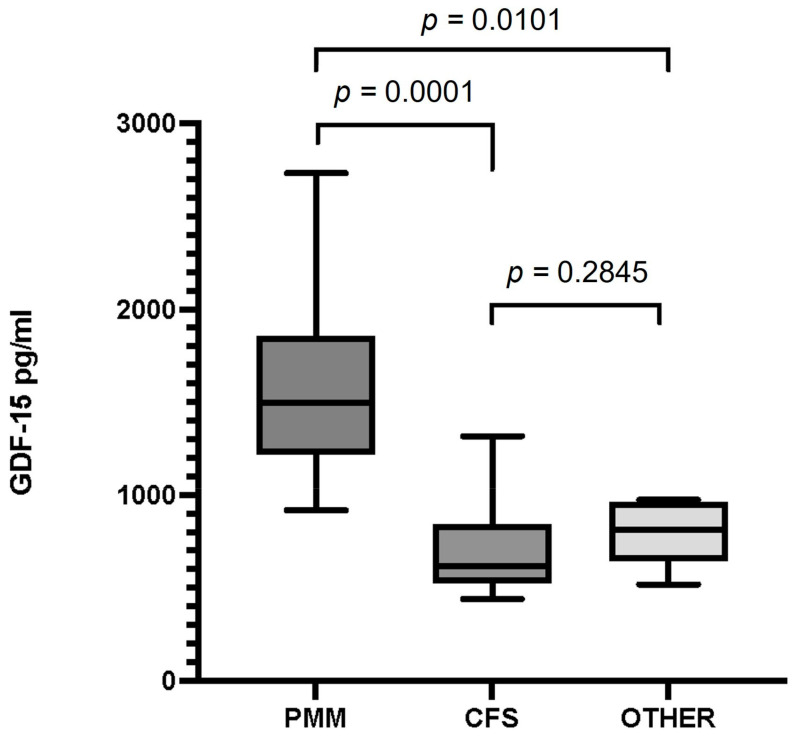
Serum GDF-15 levels in patients with primary mitochondrial myopathy (PMM), chronic fatigue syndrome (CFS), and patients with fatigue and a non-mitochondrial disorder (Other). Data are presented as the median, interquartile range, minimum, and maximum. Mann–Whitney test *p*-values are indicated on top of the figures. Significant differences (*p* < 0.05) were observed between GDF-15 levels in the PMM and CFS groups and between PMM and the group of other disorders. There were no differences between GDF-15 levels in patients with CFS and the group of other disorders.

The median value of serum GDF-15 levels (interquartile range) in patients with PMM was 1498 pg/mL (916–1856), 618 pg/mL (440–815) in patients with CFS, and 814 pg/mL (518-948) in patients with fatigue due to a non-mitochondrial disorder. We found statistically significant differencesbetween mean GDF-15 levels in patients with PMM and those inpatients with CFS, and also between patients with PMM and in patients with non-mitochondrial disease (*p* = 0.0001 and *p* = 0.0101, respectively). We found no statistically significant differences (*p* = 0.28) when comparing the mean levels of patients with CFS to those of patients with other disorders presenting with fatigue of non-mitochondrial origin (Figure 1).

**Figure 2:**
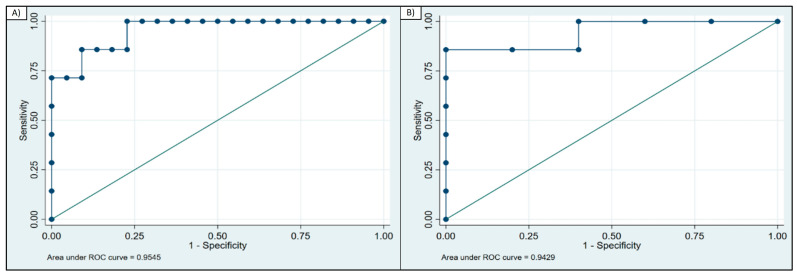
ROC curve analysis of GDF-15. (**A**) ROC curve for GDF-15 calculated for patients with PMM and CFS (AUC = 0.95); (**B**) ROC curve for GDF-15 calculated for PMM and patients with non-mitochondrial disorder (AUC = 0.94).

The diagnostic ability of GDF-15 to distinguish patients with PMM from patients with CFS, based on ROC curve analysis, showed an area under the curve (AUC) = 0.95 (95% CI 0.88–1.00) (Figure 2a). A GDF-15 concentration above 1214 pg/mL diagnosed PMM vs. CFS with 86% sensitivity and 91% specificity (correctly classified 89% of the patients).

When assessing the ability to discriminate between patients with PMM and patients with a non-mitochondrial disorder, the AUC = 0.942 (95% CI 0.81–1.00) (Figure 2b). A GDF-15 concentration above 1097 pg/mL correctly identified patients with PMM vs. the group of other non-mitochondrial disorders with 86% sensitivity and 100% specificity. 

## 4. Discussion

The usefulness of GDF-15 levels for diagnosing patients with mitochondrial disease, particularly in cases with skeletal muscle involvement, has been widely demonstrated in previous reports [8,10,11,12,13]. However, its use has not yet been transferred to standard clinical practice. In this study, we have demonstrated its ability to distinguish which patients with non-specific symptoms, such as fatigue and exercise intolerance, may have an OXPHOS disorder, aiding in selecting patients who should undergo more extensive studies. We only tested GDF-15, which has a higher diagnostic yield [6]. We used a commercial kit with an automated method and standardized control values to simplify the analysis and facilitate its use in routine clinical practice. Serum GDF-15 levels showed excellent discriminatory ability in distinguishing patients with PMM from patients with other causes of fatigue (AUC > 0.9). 

Although possible, it is rare for patients with PMM to present exclusively with exercise intolerance or fatigue without other symptoms or clinical signs suggestive of mitochondrial pathology (ptosis, ophthalmoparesis, muscle weakness, hearing loss, etc.); however, when faced with a CFS patient presenting exclusively with non-specific muscular symptoms and a normal neurological examination, a mitochondrial disease often enters the differential diagnosis, leading to extensive workup to rule it out. The diagnosis of CFS is clinical, but excluding other conditions with similar symptoms is recommended [14]. In this sense, no established protocols standardize which tests should be performed to rule out other pathologies, and patients are studied heterogeneously. In some cases, a normal physical examination, normal electromyography (EMG), and normal CK levels are considered sufficient to rule out a primary muscle disorder. Still, at other times, a muscle biopsy or genetic testing is performed to rule out this possibility.

Elevated CK levels are a valuable marker for distinguishing patients with primary myopathies from patients with CFS. However, normal CK levels do not exclude a muscle disorder, especially in the case of mitochondrial myopathies, which can present with normal or only slightly elevated CK levels. An exception are the mtDNA depletion/multiple deletions syndromes with muscle involvement, such as TK2 or *POLG*-related disorders where CK can be very high [15]. Conversely, mildly altered CK levels do not always indicate a primary muscle disease and may be present in patients with fatigue of another origin (e.g., sleep apnea-hypopnea syndrome [16], toxic myopathies, etc.) [17].

EMG results may also be misleading. An EMG reported as myopathic does not always indicate the presence of an underlying muscle disorder and should always be interpreted with caution and analyzed in conjunction with the results of the additionally performed studies. Indeed, many of the patients in our series who were ultimately diagnosed with CFS had undergone an EMG reported as pathological and had been diagnosed with a probable myopathy based solely on this finding, which was later proved wrong after additional studies were carried out.

Muscle biopsy results from patients diagnosed with mitochondrial myopathy showed clear signs of oxidative phosphorylation dysfunction (OXPHOS) in all cases, including the presence of RRF and COX-negative fibers. On the contrary, these alterations were not found in muscle samples from patients with chronic fatigue syndrome (CFS). Muscle biopsy is a highly sensitive diagnostic tool for patients with mitochondrial disease and muscle symptoms and has been widely used to reach the diagnosis [18,19]. However, being an invasive procedure, it is crucial to have a biomarker that can accurately identify the patients who will benefit the most from undergoing it. The results of our study demonstrate that GDF-15 may help distinguish which patients have an underlying mitochondrial disease in the context of a nearly normal neurologic examination and symptoms that may be confused with those of patients with CFS. This makes GDF-15 a valuable diagnostic tool in such clinical scenarios, reinforcing previous findings about its usefulness in diagnosing mitochondrial disorders.

It should be noted that although GDF-15 will help to avoid unnecessary biopsies in patients with CFS, in many cases, biopsy performance is still necessary for the definitive diagnosis of PMM in adults. Identifying the underlying genetic alteration in PMM sometimes requires the analysis of mtDNA extracted from the skeletal muscle to detect large-scale single deletions, somatic variants, or mtDNA variants with low mutation loads in other tissues, such as blood or uroepithelial cells [20]. On other occasions, the study of muscle mtDNA allows the detection of multiple deletions that guides the diagnosis towards disorders of mtDNA maintenance, which frequently present with muscle symptoms [15,21].

## 5. Conclusions

GDF-15 is a useful serum biomarker that helps distinguish between patients with chronic fatigue syndrome and those with mitochondrial myopathies exhibiting near-normal neurological examination (AUC > 0.9). Its use in this context can aid in determining which patients require further testing for a potential underlying mitochondrial disorder, thus avoiding unnecessary tests in those who will not benefit from them.

## Figures and Tables

**Table 1:** Clinical characteristics and complementary studies of the 34 patients included.

Category	ID	Age (y)	Sex	Genetic Diagnosis/Genetic Test	Muscle Biopsy	Respiratory Chain Activity	CK (UI/L) N < 170	Lactate (mmol/L) N< 2.5	GDF-15 * (pg/mL) [Reference Value, Median, P95]	Electrophysiological Study
Mitochondrial myopathy, manifested as exercise intolerance and fatigue	1	37	F	*MTCO1*: m.5992G>A, p.(Gly30Asp), (44% HET, in muscle)	RRF (10%), COX-negative fibers	N	161–1000	3	1221 [500, 852]	NA
	2	42	M	*POLG*: c.2573C>T, p.(Thr858Ile), heterozygous	RRF (8), COX-negative fibers	N	345	1.2	917 [614, 1229]	Myopathic
	3	53	F	*POLG*: c.2864A>G, p.(Tyr955Cys), heterozygous	RRF (3%), COX-negative fibers	N	67-281	1.9	1498 [757, 1466]	Sensory axonal PNP
	4	51	F	*POLG*: c.2573C>T, p.(Thr858Ile), heterozygous	RRF, COX-negative fibers	Multiple complex deficiencies	380–1379	3	1417 [757, 1466]	NA
	5	40	M	*MTTL1*:m.3243A>G, (80% HET, urine)	NA	NA	159–574	2.5	1856 [614, 1229]	NA
	6	58	M	*MTTK*: m.8433A>G, (21% HET, muscle)	RRF (20%), COX-negative fibers	Complex I and IV deficiency	153–419	2.2	2734 [757, 1466]	Neurogenic
	7	30	M	*TK2*: c.323C>T, p.(Thr108Met), homozygous	RRF, COX-negative fibers (4%). Mild myopathic changes	N	564–5344	1.5	1718 [500, 852]	NA
Other myopathies, manifested as exercise intolerance and fatigue	8	52	F	*ANO5*: c.692G>T, p.(Gly231Val), homozygous	N	N	455–2425	NA	518 [757, 1466]	N
	9	47	F	*CAPN3*: c.1714C>G, p.(Arg572Gly), heterozygous	N (partial calpain deficit)	N	54–3075	1.9	948 [614, 1229]	NA
	10	57	M	*ANO5*: c.191dupA, p.(Asn64fs), homozygous	Mild, unspecific changes	NA	1427–1800	3.3	973 [757, 1466]	NA
	11	39	M	*CAPN3:* c.1714C>G, p.(Arg572Gly), heterozygous	N	N	778–3616	2.4	814 [500, 852]	N
	12	29	M	*RAPSN:* [c.1185del] + [c.264C>A], [p.(Thr396fs)] + [p.(Asn88Lys)]	N	NA	158	1.7	770 [500, 852]	Altered RNS
Chronic fatigue syndrome (CFS)	13	58	F	NA	N	N	49	0.9	1029 [757, 1466]	Myopathic
	14	43	F	Negative/(A, B, D)	N	N	131	0.7	1317 [614, 1229]	NA
	15	59	F	Negative/(B, C, E)	Mild, unspecific changes	NA	95	2.1	616 [757, 1466]	N
	16	43	F	Negative/(E)	N	N	84	1.6	509 [614, 1229]	N
	17	54	F	Negative/(A, C)	N	N	74	1.1	671 [757, 1466]	N
	18	56	F	Negative/(A, C, D)	N	NA	94	1.1	718 [757, 1466]	N
	19	60	M	Negative/(C)	Mild, unspecific changes	N	147	0.9	571 [866, 1476]	NA
	20	50	F	Negative/(C)	N	N	97	2.2	634 [757, 1466]	N
	21	64	M	Negative/(A, C)	N	N	N	NA	926 [866, 1476]	N
	22	46	F	Negative/(A, C, D)	N	Complex I deficiency	141	2.3	505 [614, 1229]	N
	23	46	F	NA	N	N	45	NA	626 [614]	NA
	24	45	F	Negative/(B, E)	Mild, unspecific changes	NA	49	1.9	1267 [614, 1229]	N
	25	40	F	Negative/(A, C)	N	N	53	1.5	530 [614, 1229]	NA
	26	41	F	Negative/(C)	N	N	97	1.9	500 [614, 1229]	Myopathic
	27	41	F	NA	N	NA	51	1.9	582 [614, 1229]	Myopathic
	28	49	F	Negative/(C)	N	N	98	1.6	563 [614, 1229]	N
	29	52	F	Negative/(D)	N	NA	12	1.2	815 [757, 1466]	N
	30	36	F	NA	N	NA	48	0.9	480 [500, 852]	Myopathic
	31	55	F	Negative/(C)	N	N	43	1.5	1206 [757, 1466]	Myopathic
	32	28	M	Negative/(C)	N	N	174	NA	621 [500, 852]	NA
	33	48	F	Negative/(A, B)	N	N	71	0.6	536 [614, 1229]	N
	34	23	F	Negative/(C, D)	N	N	61	NA	440 [500, 852]	N

CFS: chronic fatigue syndrome, F: female, M: male, N: normal, NA: not available, Y: years, PNP: polyneuropathy, RNS: repetitive nerve stimulation, RRF: ragged-red fibers, HET: heteroplasmy. Genetic test: A: mtDNA single or multiple deletions assessment (Southern blot and/or long-range PCR), B: whole mtDNA sequencing, C: most frequent mtDNA point mutations and POLG analysis, D: NGS panel of genes related to metabolic myopathies. E: NGS panel of nuclear genes involved in mtDNA maintenance. Gene transcripts: *POLG* (NM_002693.3), *TK2* (NM_004614.5), *ANO5* (NM_213599.3), *CAPN3* (NM_000070.3), *RAPSN* (NM_005055.5). * GDF-15, kit manufacturer reference values according to age (Roche).

## Data Availability

The datasets analyzed during the current study are available from the corresponding author on reasonable request.

## Supplementary Materials

The following supporting information can be downloaded at: https://www.mdpi.com/article/10.3390/jcm12062435/s1, Table S1: Mutations and genes included in each genetic panel.
