# Supplementary material for: Serum GDF-15 Levels Accurately Differentiate Patients with Primary Mitochondrial Myopathy, Manifesting with Exercise Intolerance and Fatigue, from Patients with Chronic Fatigue Syndrome
Source: J Clin Med. 2023 Mar 22;12(6):2435. doi: 10.3390/jcm12062435 (PMC10059275; doi:10.3390/jcm12062435)
Supplement: Supplementary file 1 [file jcm-12-02435-s001.zip › jcm-2254996-supplementary.pdf]

Supplementary Table 1. Mutations and genes included in each genetic panel.

Most common pathogenic variants in mtDNA and *POLG*
MT-RNR1 m.1555A>G<br>*MT-TL1* m.3243A>G<br>*MT-ND1* m.3460G>A (p.Ala52Thr)<br>*MT-TK* m.8344A>G<br>*MT-ATP6* m.8993T>G (p.Leu156Arg)<br>*MT-ATP6* m.8993T>C (p.Leu156Pro)<br>*MT-ATP6* m.9176T>C (p.Leu217Pro)<br>*MT-ATP6* m.9176T>G (p.Leu217Arg)<br>*MT-ND3* m.10158T>C (p.Ser34Pro)<br>*MT-ND3* m.10191T>C (p.Ser45Pro)<br>*MT-ND4* m.11777C>A (p.Arg340Ser)<br>*MT-ND4* m.11778G>A (p.Arg340His)<br>*MT-ND4* m.11832G>A (p.Trp358*)<br>*MT-ND5* m.13513G>A (p.Asp393Asn)<br>*MT-ND5* m.13514A>G (p.Asp393Gly)<br>*MT-ND6* m.14459G>A (p.Ala72Val)<br>*MT-ND6* m.14482C>A (p.Met64Ile)<br>*MT-ND6* m.14482C>G (p.Met64Ile)<br>*MT-ND6* m.14484T>C (p.Met64Val)<br>*MT-ND6* m.14487T>C (p.Met63Val)<br>*POLG* c.1399G>A (p.Ala467Thr)
mtDNA maintenance panel
DGUOK, MFN2, MPV17, OPA1, POLG, POLG2, RRM2B, SLC25A4, SUCLA2, SUCLG1, TK2, TWNK, TYMP
Metabolic myopathies panel
ABHD5, ACADL, ACADM, ACADS, ACADVL, AGL, ALDOA, AMPD1, ATP2A1, CPT1B, CPT2, ENO3, ETFA, ETFB, ETFDH, GBE1, GYG1, GYS1, HADHA, HADHB, LDHA, LPIN1, PFKM, PGAM2, PGM1, PGK1, PHKA1, PHKB, PNPLA2, PYGM, SLC22A5, SLC25A20
